# Intranasal and oral vaccination with protein-based antigens: advantages, challenges and formulation strategies

**DOI:** 10.1007/s13238-015-0164-2

**Published:** 2015-05-06

**Authors:** Shujing Wang, Huiqin Liu, Xinyi Zhang, Feng Qian

**Affiliations:** Department of Pharmacology and Pharmaceutical Sciences, School of Medicine and Collaborative Innovation Center for Diagnosis and Treatment of Infectious Diseases, Tsinghua University, Beijing, 100084 China

**Keywords:** mucosal vaccine, protein antigen, adjuvant, immunostimulant, vaccine delivery system

## Abstract

Most pathogens initiate their infections at the human mucosal surface. Therefore, mucosal vaccination, especially through oral or intranasal administration routes, is highly desired for infectious diseases. Meanwhile, protein-based antigens provide a safer alternative to the whole pathogen or DNA based ones in vaccine development. However, the unique biopharmaceutical hurdles that intranasally or orally delivered protein vaccines need to overcome before they reach the sites of targeting, the relatively low immunogenicity, as well as the low stability of the protein antigens, require thoughtful and fine-tuned mucosal vaccine formulations, including the selection of immunostimulants, the identification of the suitable vaccine delivery system, and the determination of the exact composition and manufacturing conditions. This review aims to provide an up-to-date survey of the protein antigen-based vaccine formulation development, including the usage of immunostimulants and the optimization of vaccine delivery systems for intranasal and oral administrations.

**Electronic supplementary material:**

The online version of this article (doi:10.1007/s13238-015-0164-2) contains supplementary material, which is available to authorized users.

## INTRODUCTION

As the most effective way to reduce diseases, vaccination has undergone a long way in human history. Ever since 1796 when Edward Jenner used cowpox virus vaccine to prevent smallpox, vaccination has been widely used in diseases including small pox, diphtheria, tetanus, yellow fever, pertussis, *Haemophilus influenza* type b disease, poliomyelitis, measles, mumps, rubella, typhoid, rabies, anthrax, rotavirus, shingles, meningococcal, pneumococcal disease, Japanese encephalitis, varicella, rotavirus, lyme disease, tuberculosis, hepatitis and influenza (Jariyapong et al., 2013). The vaccine development evolves from natural exposure, to empirical inactivated/attenuated pathogens, and finally to subunit antigens that are structure-function properly designed nowadays (Dormitzer et al., 2012; De Gregorio and Rappuoli, 2014).

Most pathogens initiate their infections at the mucosal surface of the respiratory, gastrointestinal and urogenital systems (Marasini et al., 2014). As the first defense line for human body, mucosal immunity is highly desirable to provide an efficient and long-lasting protection against pathogen invasion. Yet, most commercial vaccines are delivered systemically, which only induces humoral immune protection without pathogen-specific mucosal immunity. Therefore, mucosal vaccination is highly advantageous for infectious diseases that is inhaled, ingested or sexually transmitted such as influenza (Tamura and Kurata, 2004), coronaviruses (Liu et al., 2011), HIV (Rappuoli and Aderem, 2011), etc. The reader is referred to several reviews describing the mucosal vaccine development against diverse infectious diseases and even cancers (Holmgren and Czerkinsky, 2005; Neutra and Kozlowski, 2006; Lycke, 2012).

Generally, several factors should be considered for an efficient and safe mucosal vaccine development, including the antigen, adjuvant, formulation, administration route and animal model for efficacy and safety evaluation. An effective vaccine often contains the following components: 1) antigens for eliciting specific adaptive immune response; 2) immunostimulants to stimulate the innate immune system and 3) delivery systems for the right-place and right-time vaccine delivery (Pashine et al., 2005). Although virus- and DNA-based antigens may be more effective, safety concerns remain due to the existence of gene-coding materials, which may revert to virulent disease-causing states. Protein antigens present a quite promising alternative for vaccine development, due to the following characteristics: 1) absence of infectious materials like coding genes, 2) capability of inducing antigen-specific antibodies, 3) possibility for chemical modification and 4) the readiness for large scale manufacturing for a looming pandemic. However, most protein-based antigens have the limitation of physiological instability and low immunogenicity, which demand both potent immunostimulants and efficient delivery systems to accomplish effective vaccine products.

Here, we review the up-to-date achievement of mucosa prophylactic vaccine development with protein-based antigens to defend against various infectious diseases, including tetanus, influenza, hepatitis, SARS, MERS, HIV, etc. The field is vast and this review merely concentrates on the recent, relevant and most studied protein antigens, adjuvants and delivery systems for oral and intranasal vaccinations.

## IMMUNOLOGICAL AND BIOPHARMACEUTICAL ASPECTS OF INTRANASAL AND ORAL VACCINATION

Vaccines are delivered through various administration routes, including parenteral routes like intramuscular or subcutaneous injection, and mucosal routes through intranasal, oral, vaginal or rectal tract. Mucosal vaccination has several foreseeable advantages: 1) needle free and better patient compliance; 2) strong mucosal immunity besides systemic immune responses, which provides the first barrier against those infections initiating at the mucosal surface; 3) potential to overcome the barrier of the pre-existing immunity caused by previous parenteral vaccinations (Belyakov et al., 1999).

Intranasal and oral vaccinations are the most attractive administrative routes among various mucosal administrations, largely due to their better patient compliance. Nasal delivery is preferred due to: 1) the highly vascularized mucosal surface area of 150 cm^3^ from the naso-pharyngeal compartment for vaccine uptake, 2) the ability to induce immune protection at local nasal, interconnected oral and distant mucosal sites such as vaginal and colorectal regions, and 3) relatively low dose to achieve required immunity, compared with other routes (Almeida and Alpar, 1996; Olszewska and Steward, 2001; Holmgren and Czerkinsky, 2005). Oral delivery is advantageous considering its superior patient compliance, easy administration and mass immunization capacity (Marasini et al., 2014), especially when it comes to the plant-derived protein antigens and veterinary vaccines. For example, plant-derived recombinant protein vaccines are more efficient and cost effective for oral administration, without protein purification or complicated formulating steps as algae-based oral recombinant vaccines (Specht and Mayfield, 2014). Meanwhile, plant-derived protein antigens are suggested to be used as boosting vaccines by just orally feeding animals with the antigen-expressing food, where the priming can be realized with conventional vaccinations (Lamphear et al., 2004; Pogrebnyak et al., 2005).

**Figure 1:**
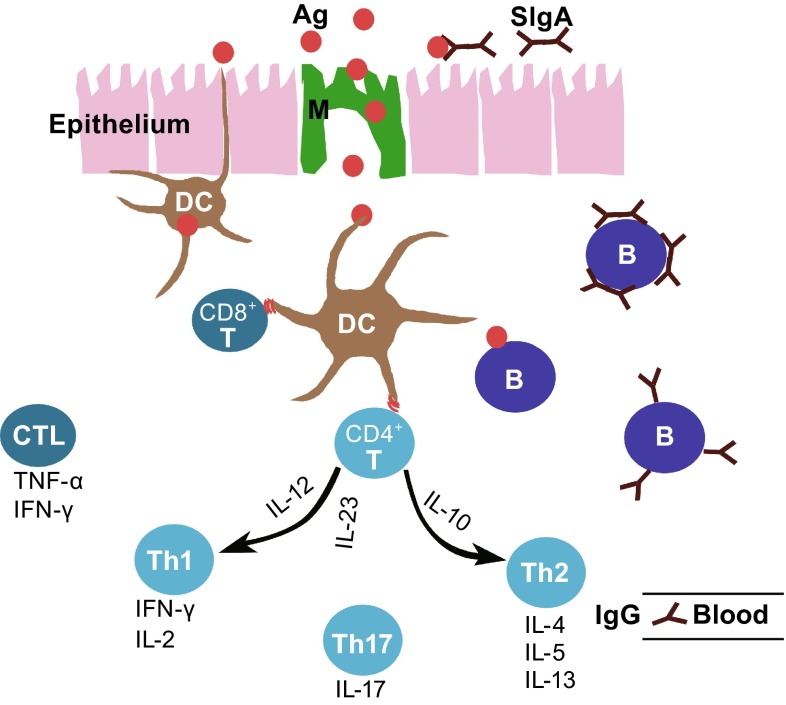
**Schematic illustration of mucosal immunity**. Ag: antigen; M: M cells; SIgA: secretory IgA

The mucosal surface is protected by the large and specialized innate and adaptive mucosal immune system. Innate immune system plays an important role in fighting against initial infections and facilitating generation of adaptive immune response, while adaptive immune system is vital for providing protection against previously encountered pathogens. The mucosal immunization occurs at the inductive sites called the mucosa-associated lymphoid tissue (MALT), which contains B cells, T cells and antigen presenting cells (APCs) for specific immune response initiation (Holmgren and Czerkinsky, 2005; Lawson et al., 2011). The MALT is covered by a follicle-associated epithelium, comprising epithelium cells, lymphoid cells and a minor portion of microfold/membraneous (M) cells (Fig. 1). M cells are generally recognized as the antigen uptaking cells from the lumen of intestinal/nasal mucosa and transport antigens to the underlying APCs in MALT. Upon infection or vaccination, precursor cells (B cell, T cell and dendritic cells) in inductive sites can be activated, and then migrate and populate the local or remote mucosa sites through the common mucosal immune system to realize the systemic immune protection. Most mucosal response occurs at the local initiation and adjacent interconnected mucosa. As an exception, intranasal vaccination could induce IgA secretion not only at local nasal and adjacent oral mucosa surface, but also remote vaginal and rectal regions (Holmgren and Czerkinsky, 2005).

Similar to systemic immunization, mucosal vaccination can be accomplished through three steps (Fig. 1): 1) efficient antigen sampling and uptake; 2) antigen processing and presentation by APCs; and 3) B and T cells activation, production of effector cells and generation of their memory counterparts. The production of neutralizing antibodies is vital for humoral immune response in clearing extracellular infections. Distinguished from systemic vaccination, mucosal vaccination normally induces strong secretory IgA response to defend the viral infection at the mucosal surface, besides producing systemic serum IgG to neutralize the newly generated viruses (Renegar et al., 2004). Cellular immune responses involve the activation of CD4^+^ and CD8^+^ T cells. CD4^+^ T cells can activate and differentiate into different functional effector cells: type-1 (Th1), type-2 (Th2) and type-17 (Th17) (Khader et al., 2009). The production of Th1 is induced by interleukin-12 (IL-12), and Th1 effector cells can produce interferon-γ (IFN-γ) and mediate cellular response against intracellular pathogens. Th2 effector cells produce IL-4 and regulate humoral immune responses. The Th17 effector cells are generated in the presence of IL-23, characterized by the production of IL-17 and critically involved in the defense against pathogens at the mucosa surfaces (Khader et al., 2007; Khader et al., 2009). Activated CD8^+^ T cells (cytotoxic T lymphocytes, CTLs) have cytolytic effect and also mediate the production of cytokines like interferon-γ (IFN-γ) and tumor necrosis factor-α (TNF-α) (Seder and Hill, 2000). CTL responses are critical for the protection against intracellular infections. An optimal mucosal vaccine formulation, including the appropriate combination of antigens, immunostimulants and delivery carriers, should be able to induce a comprehensive series of protective immune response, as demonstrated by the production of various antibodies (IgG, IgA, etc.), Th1, Th2, Th17, CTLs and relevant cytokines (Fig. 1).

Pathogen microbes often have specific molecular characteristics known as pathogen-associated molecular patterns (PAMPs), which can be recognized by pathogen recognition receptors (PRRs) on mucosal epithelial cells and APCs. PRRs are generally membrane-bound receptors such as toll-like receptors (TLRs), nucleotide-binding and oligomerization domain (NOD)-like receptors (NLRs), and C-type lectin receptors (CLRs) (Kanzler et al., 2007; Devriendt et al., 2012; Park, 2014). The sensory of PAMPs on pathogens by PRRs on DCs is passed to T and/or B cells through altering the release of cytokines, the expression of co-stimulatory molecules and the up-regulation of integrins for adhesion. To improve the potency of vaccines, immunostimulants targeting these PRRs are often used as adjuvants to activate and prepare the immune system for reacting to specific antigens. Immunostimulants could be PAMPs or their derivatives, endogenous cytokines and other empirical molecules or materials, details to be discussed in section ADJUVANTS FOR MUCOSAL VACCINES WITH PROTEIN ANTIGENS.

Before antigens being processed and presented by APCs, the first requirement in mucosal vaccination is to guarantee efficient antigen transportation through the mucosal epithelium from the mucin to the MALT. This is realized by endocytosis (receptor-mediated or not) of antigens at the apical membrane and exocytosis at the basolateral membrane by M cells and other enterocytes (Neutra et al., 1996; Lawson et al., 2011; Reineke et al., 2013). One way to improve the transcytosis of vaccine by M cells or other epithelial cells is to incorporate the M/epithelial cell-specific ligands or pathogen-exploited molecules in the vaccine formulation. Once the vaccine reaches the MALT underlying the mucosa epithelium, efficient uptake, processing and presentation of antigens are demanded for APCs including macrophages, dendritic cells (DCs) and B cells. DCs, as the key APCs that bridging the innate and adaptive immune systems, are found to prefer the intake of vaccines formulated into pathogen-like nanoparticles (Elamanchili et al., 2007; Klippstein and Pozo, 2010; Hamdy et al., 2011). Thus, quite a number of nanoparticular vaccine delivery systems based on different biomaterials have been explored to target the PRRs on DCs and mimick the pathogen properties to enhance immune responses (Demento et al., 2011; Park, 2014). Mature DCs can then potently activate the naïve T cells and act as the primary initiator of the immune response against specific antigens (Banchereau and Steinman, 1998). All the aforementioned properties and functions of epithelial cells, M cells and DCs are widely exploited in various mucosal targeting strategies as discussed in section VACCINE FORMULATIONS: MATERIALS AND DELIVERY SYSTEMS.

## PROTEINS AS AN EMERGING CLASS OF ANTIGENS

Antigens are the central elements of vaccines, which are used to induce the antigen-specific immune memory. Currently, there are various types of antigens, including 1) whole inactivated pathogens or mixtures; 2) live-attenuated organisms; 3) vector-based recombinant vaccines; 4) subunit vaccines including DNA, RNA, isolated/recombinant proteins, glycoproteins and carbohydrates. Most commercially available vaccines fall into the first and second categories containing genetic materials of pathogens, with only a few exceptions that include protein-based subunits such as virus-like particles (VLPs), detoxified toxoids/toxins and polysaccharide-protein conjugates (http://www.fda.gov/BiologicsBloodVaccines/Vaccines). Approved vaccines are generally administered systemically, except for a few mucosal vaccines comprising whole inactivated or live-attenuated pathogens, such as intranasal influenza vaccine (FluMist from MedImmune, LLC) and oral vaccines against polio (oral polio vaccine), cholera (oral cholera vaccine), typhoid fever (Vivotif), adenovirus (no trade name from Barr Labs, Inc) as well as rotavirus infections (ROTARIX from GlaxoSmithKline Biologicals).

Live viral vectors are widely used as delivery systems in mucosal vaccination, including adenovirus, attenuated influenza virus, Venezuelan equine virus, bacillus Calmette-Gue´rin and poxvirus vectors (Prevec et al., 1989; Caley et al., 1997; Hiroi et al., 2001; Gherardi and Esteban, 2005; Huang et al., 2009; Wang et al., 2009). Besides viral vectors, nucleic acid-based vaccines such as plasmid DNA and RNA, are also being developed. However, the inactivated/attenuated pathogens, vector- and DNA-based vaccines are always limited for use due to difficulties in microorganism culturing and some safety concerns such as the possibility of reverting to the virulent state in immunocompromised hosts, as well as potential adverse effects including allergic and autoimmune reactions. In contrast, vaccines with protein antigens are intrinsically safer than the whole pathogen-based and DNA-based antigens due to the absence of genetic materials. Furthermore, the technical maturation in mass production of specific peptides and recombinant proteins has substantially lowered the hurdle of developing protein/peptide-based therapeutics and vaccines (Degim and Celebi, 2007). Therefore, pathogen proteins and epitope peptides provide a promising alternative for antigen development. For example, the Nabel group reported that ferritin nanoparticle based HA delivery system demonstrated a more potent and broader immune protection against influenza than the inactivated virus. In this vaccine delivery system, HA mimics its natural trimeric conformation as in the virus (Kanekiyo et al., 2013).

Currently there have not been any approved oral or intranasal protein vaccines yet, but extensive efforts have been reported on mucosal vaccination with protein-based antigens against various infectious diseases such as influenza (Yoshikawa et al., 2002; Tamura and Kurata, 2004; Petersson et al., 2010; Rose et al., 2012), plaque (Eyles et al., 1998; Tripathi et al., 2006), tetanus (Jaganathan et al., 2005), diphtheria (Alpar et al., 2001; Singh et al., 2006), hepatitis B (Borges et al., 2007; Borges et al., 2008), HIV (Morris et al., 2000), SARS-CoV (Pogrebnyak et al., 2005) and MERS-CoV (Zhang et al., 2014), etc. Besides proteins from pathogens, model proteins antigens such as ovalbumin (OVA), β-galactase (β-gal) and bovine/human serum albumin (BSA/HSA) are often used in the vaccine formulation development.

Influenza viruses infect host cells through two surface glycoproteins: hemagglutinin (HA, of a head region HA1 and a highly conserved stalk region HA2) and neuraminidase (NA). Annual influenza pandemics occur as the antigenic properties of HA and NA changes. Thus, both proteins serve as potential candidates as influenza vaccine antigens for intranasal immunization (Yoshikawa et al., 2002). On the other hand, the matrix proteins (M1 & M2), whose amino acid sequences are highly conserved among human influenza A viruses, are exploited to design broad-spectrum influenza vaccines (Mould et al., 2000; Sui et al., 2010).

Envelope surface glycoprotein 120 (gp120) and transmembrane glycoprotein 41 (gp41) of HIV mediate HIV infection by interacting with the CD4 receptors of the host cell. Thus, these two proteins and their short sequence fragments are often used as antigens for HIV vaccine development (Lema et al., 2014).

Tetanus toxoid (TT) is a 150 kDa protein produced by bacterium *Clostridium tetani*, which causes tetanus. Diphtheria toxoid (DT) is a 535-amino acid protein secreted by the pathogen bacterium *Corynebacterium diphtheria*, which causes diphtheria. Both toxins as the main disease-causing contributors are exploited as antigens in the vaccine development against tetanus and diphtheria (Alpar et al., 2001).

Fraction 1 (F1) capsular protein of 17.5 kDa and virulence (V) protein of 35 kDa are virulent subunits produced by *Yesinia pestis* which causes the plaque disease. Vaccines based on F1 and V protein display protective immunity in both bubonic and pneumonic animal models (Williamson and Oyston, 2013).

The surface antigen of the hepatitis B virus (HBsAg) is a viral envelope protein. It has been successfully used in human vaccines to induce effective immune protection against hepatitis B (Krugman, 1982; McAleer et al., 1984). HBsAg assembles into VLPs after recombinant expression in hosts like yeasts (McAleer et al., 1984).

There are two novel coronaviruses emerged in this century: severe acute respiratory syndrome coronavirus (SARS-CoV) and Middle East respiratory syndrome coronavirus (MERS-CoV). Both cause acute respiratory distress syndromes and lead to high mortality. The receptor binding domain of the spike protein of SARS-CoV/MERS-CoV is a promising antigen candidate since it binds to the human host-cell receptor, angiotensin-converting enzyme 2/dipeptidyl peptidase 4 (Li et al., 2003; Wong et al., 2004; Graham et al., 2013; Lu et al., 2013; Zhang et al., 2014). Orally feeding mice with tomato juice expressing the N-terminal fragment of SARS-CoV spike protein induce SARS-CoV-specific IgA production (Pogrebnyak et al., 2005).

Different protein antigens can be fused to generate combined vaccine antigens against two or more diseases at the same time. For instance, enhanced and cross protective immunity against both influenza and respiratory syncytial virus (RSV) is realized by intramuscular vaccination with fusion subunit protein of influenza virus hemagglutinin (HA) and RSV fusion (F) protein (Turner et al., 2013). Protein antigens are widely exploited in vaccine development to protect against infectious diseases, but they are seriously limited by the generally low stability and immunogenicity to induce concerted humoral and cellular immune responses. Thus, an optimal formulation (including the selection of immunostimulants) and an ideal delivery route are critically important for protein-based vaccines.

## ADJUVANTS FOR MUCOSAL VACCINES WITH PROTEIN ANTIGENS

Subunit protein antigens are much safer alternatives to the whole pathogen or viral vector-based antigens since they have lower risk of causing diseases, but the immunogenicity is often compromised at the same time. This necessitates the use of adjuvants in protein vaccines to improve the humoral, cellular and mucosal immune responses (Marciani, 2003). Adjuvants in this review specifically refer to immunostimulants or immune-potentiators, substances that stimulate the immune system by activating or increasing the activity of its components.

Immunostimulants are compounds or macromolecule complexes that can boost, maintain and potentiate the immune response of antigens (Garcia and De Sanctis, 2014). They are generally used to target the innate immune response, which further facilitate the evolution of the adaptive immune response (Pashine et al., 2005; Reed et al., 2009) and mediate the balance between humoral and cellular immunity (Brunner et al., 2010). The immunostimulating contribution of immunostimulants in a vaccine is affected by the antigen, the formulation and the administration routes. For example, TLR agonists (including FSL-1, poly I:C, CpG B, MPL/LPS, Pam3CSK4 and R848), NLR ligand (such as muramyl dipeptide (MDP)), the GM1 ganglioside receptor ligand and cholera toxin B, are evaluated as sublingual, nasal and intradermal adjuvants for HIV gp140, TT and OVA vaccine in mice. It is suggested that different adjuvants show different immunological effects upon administration through different routes (Bal et al., 2012; Buffa et al., 2012).

Adjuvants are considered as components of vaccine products and are not licensed separately. Currently licensed adjuvants, including alum (aluminum sulfate or phosphate), MF59 (squalene droplets with two surfactants), AS04 (aluminum hydroxide and monophosphoryl lipid A), AS03 (squalene, tween 80 and α-tocopherol) and virosomes (lipid and hemagglutinin), were selected and developed empirically. Here we review adjuvants that have been studied with protein-based vaccines for intranasal and oral administration in humans/animals (summarized in Table 1 and Table S1**)**, such as cholera toxin (CT), heat-labile enterotoxin (LT), alum, MPLA, dsRNA polyriboinosinic-polyribocytidylic acid (poly I:C), surf clam microparticles, Eurocine® adjuvants, compound 48/80 (C48/80), α-galactosylceramide (α-GalCer), bis-(3',5')-cyclic dimeric adenosine/guanosine/inosine monophosphate (c-di-AMP/GMP/IMP), muramyl dipeptide (MDP) and saponin-containing botanical extracts.

**Table 1:** **Summary of promising adjuvants tested for protein antigens in this review**

Classifications	Representative adjuvants	Properties, advantages and/or disadvantages
Alum	Aluminium salts (@numerous licensed products, constituents of AS04 @Fendrix)	NLRP3 pathway, show depot effect, stimulation/prolongation of Ag uptake through APCs, well sourced safe material available for vaccine applications, efficient in generating antibody responses, but not in inducing Th1 and CTL responses.
Bacterial toxins and their derivatives	Cholera toxin (@cholera vaccines), Heat-labile enterotoxin**	PAMP, bind to GM1 gangliosides receptors on mucosal cells, generate potent and broad immune response, serve as gold standards for adjuvant potency investigation, show the safety issue of causing facial paresis after intranasal administration.
Bacterial glycolipids	LPS and its derivative MPL/MPLA (@Cervarix, Supervax, Pollinex Quattro et al., components of AS01, AS02, AS04, AS15 et al.)	PAMP, TLR pathway, activate APCs and induce cytokine cascades, induce potent humoral and cellular immunity, MPL has been proven non-toxic and used in complex formulations such as oil-in-water emulsion, liposomes and adjuvant combinations with alum and QS21.
Bacterial peptidoglycan	MDP*	PAMP, NLR pathway, induce cytokine production, induce both humoral and cellular immunity, limited to veterinary vaccines since it is too pyrogenic for human use.
Bacterial second messengers	c-di-AMP/IMP/GMP	Bind STING, induce potent humoral and cellular immune responses.
Synthetic bacterial DNA and viral dsRNA	CpG***, Poly I:C*	PAMP, TLR pathway, induce potent humoral and cellular immunity.
Virosomes	Virosomes (@Epaxal, Inflexal V)	PAMP, unknown pathway, mimic characters of virus without carrying the viral genes, highly immunogenic and also act as Ag delivery system.
Synthetic small organic molecules	Imidazoquinoline (@Aldara), C48/80, Vitamin E TPGS*, α-GalCer**	Different molecules activate the immune response through various mechanisms, induce systemic and mucosal immunity.
Plant derived molecules	Saponin (especially QS21***), Eurocine**	QS21, as a component of AS01, AS02, AS15 et al., is a potent immunostimulant to both humoral and cellular immunity.
Polymers from crustaceans’ shell	Chitosan, Surf clam microparticles	Chitosan is mucoadhesive and behaves as both the immunostimulant and the antigen delivery material.
Cytokines	IL-1, IL-12**, TNF and mutants	Regarded as less toxic since they are human innate substances.
Combinations	AS01***, AS02 ***, AS04 (@Fendrix), AS15**	AS01 (liposomes, MPL, QS21), AS02 (oil-in-water emulsions, MPL, QS21), AS04 (alum, MPL, @Fendrix), AS15 (liposomes, MPL, CpG, QS21), make use of the synergistic effects of different adjuvants.

The current status in clinical study of the adjuvants are indicated by * (phase 1), ** (phase 2), *** (phase 3), and @ (licensed product with trade names). The table only summarizes the general adjuvants in protein-based vaccines mentioned in this review. More information regarding to the detailed investigation reports can be found in the Table S1

### Alum

Alum based adjuvants have been used for around 80 years since it can elicit humoral immune response upon systemic injection. Adjuvants alum and liposomes showed synergistic effect in enhancing immune response of DT and TT after oral administration in rabbits and monkeys (Mirchamsy et al., 1996). Generally, alum is a weak immunostimulant for protein antigens in the mucosal vaccines with few successful reports (Malik et al., 2012). Moreover, it is hindered for potential use in vaccine development of intracellular pathogens and tumors due to its incapability in inducing potent Th1 and CTL responses (Marrack et al., 2009; Reed et al., 2009).

### Bacteria toxins and their detoxified derivatives

Two bacterial toxins, cholera toxin (CT, of two subunits-CTA and CTB) from various strains of *Vibrio cholerae*, the heat-labile enterotoxin (LT) from enterotoxigenic strains of *Escherichia coli* and the detoxified mutant like LTK63, are used as typical mucosal adjuvants in vaccines of protein antigens. They are known to increase the epithelium permeability, modulate the vaccine uptake by APCs and the responses of lymphocytes (Cox et al., 2006). Although the use of their non-toxic mutants as adjuvants is still attractive for improving immunogenicity of antigens (Morris et al., 2000; Moschos et al., 2004; Stephenson et al., 2006), these adjuvants have not been approved in human intranasal vaccines due to the danger of redirection of antigens to the central nervous systems and causing inflammatory responses such as Bell’s Palsy (van Ginkel et al., 2000; Mutsch et al., 2004; Lewis et al., 2009). Nevertheless, their potency as mucosal adjuvants puts them as gold evaluation standard in many vaccine development researches.

### Bacterial glycolipid

Monophosphoryl lipid (MPL), derived from the lipopolysaccharide (LPS) of *Salmonella Minnesota*, is a PAMP that can induce both humoral and cellular immune responses after systemic and mucosal vaccinations (Mata-Haro et al., 2007). LPS as the adjuvant for OVA in N-trimethyl chitosan (TMC) nanoparticles induce higher IgG1 and IgA titers after intranasal vaccination in mice than that without LPS (Bal et al., 2012). Similarly, the encapsulation of MPLA together with OVA in PLGA nanoparticle increases the antigen-specific immune responses in mice after oral administration compared to the one without MPLA (Sarti et al., 2011). MPLA is the first TLR ligand (TLR4) approved for human vaccine, as a component of AS04 in the vaccine formulation against HPV and HBV (Kanzler et al., 2007; Mata-Haro et al., 2007). Apart from AS04, MPL is also the component in several adjuvants such as AS01, AS02, AS15 and GLA-SE clinically tested in human vaccines (Maisonneuve et al., 2014).

### Bacterial peptidoglycan

Muramyl dipeptide (MDP) is a peptidoglycan constituent of both Gram-positive and Gram-negative bacteria. It is a PAMP and can activate the NLRs which in turn lead to cytokine activation. However, the use of MDP as the adjuvant has been limited to veterinary vaccines due to its pyrogenic effects in human (Lemesre et al., 2007; Maisonneuve et al., 2014). After intranasal vaccination in mice, MDP adjuvanted OVA in TMC nanoparticles induce higher IgG1 and IgA titers than that without adjuvants (Bal et al., 2012). It has been suggested that the loading efficiency of small substances like immunostimulant MDP in TMC nanoparticles may decrease when co-loaded with large, water-soluble molecules like protein OVA, presumably caused by the increased leakage of MDP through diffusion channels generated by the leakage of OVA during the formulation preparation (Mathew et al., 2014).

### Bacterial second messengers

Bacterial second messengers such cyclic di-nucleotides (e.g. c-di-AMP and c-di-GMP) are suggested as promising mucosal adjuvants. They are reported to interact with the transmembrane protein stimulator of interferon genes (STING), which then increases the production of type I interferons and further drives the adaptive immune response (Ishikawa et al., 2009; Burdette et al., 2011; Shaw et al., 2013). Studies of intranasal delivery of model protein antigens such as recombinant influenza nucleoprotein (rNP), β-Gal and OVA together with the adjuvant c-di-AMP/IMP in mice suggest them as potent mucosal adjuvants, especially when cellular immunity is desired (Ebensen et al., 2007; Libanova et al., 2010; Ebensen et al., 2011; Sanchez et al., 2014). C-di-GMP can enhance the immune response in mice for H5N1 virosomes after sublingual, intranasal and intramuscular administrations (Pedersen et al., 2011).

### Synthetic bacterial DNA and viral dsRNA

The CpG motif is considered as PAMPs since it is abundant in microbial genomes but not in vertebrates. Synthetic CpG oligonucleotide is a TLR9 agonist that can induce IL-12 production in APCs and subsequently stimulate antigen-specific Th1-mediated cellular immune responses, involving CTLs (Krieg et al., 1995; Klinman et al., 2004). It was used in clinical tests as a component of HBV and anthrax prophylactic vaccines (Kanzler et al., 2007). CpG has been suggested as a suitable adjuvant in intranasal and oral vaccination for protein antigens such as HBsAg (McCluskie and Davis, 1998), TT (McCluskie et al., 2000; Eastcott et al., 2001) and HIV peptides (Pun et al., 2009; Buffa et al., 2012) as tested in mice. Poly I:C is a synthetic analogue of double stranded viral RNA, which activates TLR3 in macrophages and DCs to further promote strong T cell priming (Trumpfheller et al., 2008). It is suggested as a potent and potential adjuvant for nasal influenza vaccine with antigen HA as evidenced by the induced comparable immune response from both CpG and the standard adjuvant CTB, and also by the cross-protection observed in mice after intranasal vaccination (Ichinohe et al., 2005). Similarly, it plays a vital role in efficiently inducing systemic and mucosal immune protection against human parainfluenza viruses (HPIVs) after intranasal vaccination in mice with oligomannose-coated liposomes (OMLs) encapsulating the full-length HA-NA (HN) protein antigen (Senchi et al., 2013).

### Virosomes

Not only small molecules, polymers and subunits of microbes can act as immunostimulants too. Virosomes assembled from membrane lipids and proteins of influenza virus can also act as vaccine adjuvants to enhance immune responses. Intranasal vaccination of mice with simian-human immunodeficiency virus-VLP (SHIV-VLP) antigens and influenza virosomes adjuvants induce comparable humoral and cellular immune response as that with the adjuvant CpG (Kang et al., 2004).

### Synthetic small organic molecules (imidazoquinoline, compound 48/80, vitamin E TPGS, glycolipid α-galactosylceramide)

Imidazoquinolines, such as imiquimod or gardiquimod, are synthetic TLR7/8 agonists. Imiquimod is able to induce balanced humoral and cellular immune responses when co-delivered with HBsAg in chitosan nanocapsules upon intranasal vaccination in mice (Vicente et al., 2013). Compound 48/80 (C48/80), a mast cell activating compound, is shown to be a safe and effective nasal adjuvant in mice co-administered with a botulimun neurotoxin A (BoNT/A) immunogen-Hcbtre and in rabbits co-administered with recombinant HA (Meng et al., 2011; Staats et al., 2011). Vitamin E TPGS (d-α**-**tocopheryl polyethylene glycol 1000 succinate), a water soluble vitamin E derivative, is suggested as a promising nasal vaccination potentiator when encapsulating the DT together with PCL and tested in mice (Somavarapu et al., 2005). Alpha-galactosylceramide is a natural glycolipid derived from murine sponge and now is mainly chemically synthesized. It can be presented by APCs to potently activate natural killer T (NKT) cells, and modulate T cell immunity through efficient activation/maturation of DCs (Kawano et al., 1997). Courtney et al. reported that repeated dosing of α-galactosylceramide intranasally or orally induced potent systemic and mucosal immune response in mice with HIV gp120 epitope peptides as antigens (Courtney et al., 2009).

### **Plant derived molecules** (**saponins and lipids)**

Saponins are plant-derived chemical compounds with various biological and pharmaceutical activities. They are amphipathic with hydrophilic glycoside moieties and lipophilic triterpene derivatives. Saponin extracts can stimulate Th1 immune response and CTL production against antigens, which suggests them as potential adjuvants for vaccines against intracellular pathogens and tumor cells. Yet, they are restricted from human vaccination usage due to their toxicity, instability and haemolytic effects. A purified fraction of QS, QS21 was used in clinical trials (Skene and Sutton, 2006; Sun et al., 2009a). The purified QS has been approved and it is commercially used in veterinary vaccines like bovine respiratory syncytial virus vaccine (Ellis et al., 2005). A semi-synthetic saponin analog GPI-0100 is a potent mucosal adjuvant to induce systemic and mucosal immune responses after subcutaneous and intranasal administration in mice for the non-fibril adhesin hemagglutinin B (HagB) (Zhang et al., 2003). In the intranasal immunization, the potency of GPI-0100 was found to be only second to LT and its mutants, while higher than other tested adjuvants such as MPLA, alum and CTB. Subunit (HA) influenza vaccines with Eurocine® adjuvants of plant lipids (mono-olein, oleic acid, lauric acid, soybean oil) could induce protective immunity upon intranasal administration in mice (Petersson et al., 2010).

### Polymers from crustaceans’ shell (e.g., chitosan) or particles from surf clam

Chitosan is a de-acetylated derivative of the polysaccharide chitin, which is extracted from crustaceans’ shells. As a mucoadhesive polymer, it prolongs the mucosal residence and increases the uptake of vaccines by APCs. Chitosan-containing vaccines have been subjected to human clinical trials, suggesting its low-toxicity (Illum et al., 2001; Moschos et al., 2004). Sui et al. reported that influenza M1 vaccine using chitosan as the adjuvant induced cross-protection against influenza virus infection after intranasal vaccination in mice (Sui et al., 2010). Surf clam microparticles (SMP), processed from surf clam shells, was reported to induce humoral and mucosal immune response when intranasally administered with influenza HA vaccine in mice (Ichinohe et al., 2006).

### Human endogenous proteins like cytokines

Interleukin (IL)-12 is a cytokine secreted by APCs upon antigenic stimulation. IL-12 can potently induce the production of IFN-γ by NK and T cells, and lead to T cell development into Th1 cells (Trinchieri, 1995). Boyaka et al. reported IL-12 as an effective adjuvant in intranasal immunization in mice with antigen TT (Boyaka et al., 1999). Cytokines of IL-1 family as adjuvants was found to be able to increase the HA-specific IgG titers in serum and IgA titers at mucosal surface after intranasal immunization in mice compared to the one without IL-1 (Kayamuro et al., 2010). Tumor necrosis factor (TNF) family members including TL1A, TNF-α and the mutant could induce antigen-specific IgG and mucosal IgA responses after intranasal administration in mice with antigen OVA or HA (Kayamuro et al., 2009). The reader is referred to the review by Wang X. et al. for more discussions on the innate endogenous adjuvants (Wang and Meng, 2014)

### Adjuvant combinations

Various adjuvants can be combined in one formulation to exploit their synergy in activating the immune system. Several adjuvants used in systemic administrations (either licensed or in clinical trials) are developed with immunostimunlants combinations such the adjuvant system series (AS01, AS02, AS04, AS15) from GlaxoSmithKline containing two or more components of alum, MPL, QS21 or CpG. Moschos et al. reported that adjuvants chitosan and the NLR ligand-MDP contribute synergistically to increase immunogenicity of recombinant *H. pylori* urease (rUre) after intranasal vaccination in mice (Moschos et al., 2004). Co-adjuvanting of c-di-GMP with chitosan showed balanced Th immune responses in mice for H5N1 vaccine with antigen HA after intranasal administration (Svindland et al., 2013).

## VACCINE FORMULATIONS: MATERIALS AND DELIVERY SYSTEMS

Unlike small molecules, proteins are macromolecules containing primary, secondary, tertiary and even quaternary structures with labile bonds and specific side-chain orientations. Harsh biological conditions (e.g., proteolytic and harsh gastric pH) could cause protein denaturation and degradation, which could further reduce their biological activities and even generate adverse immunogenicity. Therefore, the usage of delivery system and/or immunostimulants is critically important in facilitating the induction of potent and long-lasting immune protection after mucosal vaccination. For example, F1 and V subunit antigen of *Y. pestis* can induce humoral and mucosal immunity after intranasal administration in mice when co-encapsulated in PLA microsphere, or co-administrated using CTB as adjuvants, but not with free soluble F1 and V forms and in absence of adjuvants (Eyles et al., 1998).

Although the appropriate particle size for optimal mucosal vaccination remains to be determined, particulate antigens generally present more immunogenicity in mucosal vaccines than their soluble counterparts (Challacombe et al., 1992; Igartua et al., 1998; Singh and O’Hagan, 1998; Koping-Hoggard et al., 2005; Park et al., 2013; Smith et al., 2013; Marasini et al., 2014; van Riet et al., 2014; Zhao et al., 2014). Despite the limited mechanistic understanding of their behaviors *in vivo*, particulate delivery systems offer several benefits for mucosal vaccines, including: 1) prevention of antigen degradation, 2) elevated concentration of antigens in the vicinity of mucosa tissues, 3) prolonged residence and release time of vaccines, 4) co-delivery of antigens and adjuvants, 5) receptor-ligand mediated targeting delivery, and 6) acting as an immune-potentiator at the same time (Zhao et al., 2014). These functions could be optimized through adjusting their controllable properties such as sizes, geometry, surface properties, molecular patterns, antigen loading, surface decoration with functional molecules and antigen-release kinetics (Bachmann and Jennings, 2010).

Intranasal and oral administrations of vaccines using particulate delivery systems are described in several reviews (Sharma et al., 2009; Sun et al., 2009b; Marasini et al., 2014). Here we focus on materials and vehicles that are currently applied for the development of protein antigen-based vaccines (summarized in Table 2 and Table S2), including: 1) VLPs; 2) synthetic polymers such as poly lactic-co-glycolic acid (PLGA), poly lactic acid (PLA), poly ε-caprolactone (PCL), poly (ethylene glycol) (PEG) coated/conjugated copolymers like PEG-PLGA, PEG-PLA and PEG-PCL and polyethyleneimine (PEI); 3) natural polymers or their derivatives such as chitosan and alginate; 4) lipid-based vehicles, including liposomes, niosomes, bilosomes, virosomes and immune-stimulating complexes (ISCOMs); and 5) others, such as multiple antigen-presenting vaccine systems, hydrogels and inorganic vehicles such as gold nanoparticles. These materials and vehicles are explored as vaccine delivery systems to encapsulate, adsorb or conjugate protein antigens with/without adjuvants.

**Table 2:** **Summary of delivery vehicles for protein antigens discussed in this review**

Classifications	Representative materials	Properties, advantages and/or disadvantages
VLP	VLP-HBV (@GenHevac B, Engerix-B, Recombivax HB), VLP-HEV (@Hecolin), VLP-HPV (@Cervarix, Gardasil), VLP-MuPyV, VLP-NV*	Mimick the particular, ordered and repetitive structural nature of the virus, highly immunogenic, antigens can be chemically conjugated onto or genetically inserted into the VLPs, may have poor quality consistency for VLPs generated from different hosts and batches for incorporating hosts’ materials.
Synthetic polymers	PLGA, PLA, PCL, PEI and their PEGylated derivatives, Eudragits	Biocompatible, biodegradable and generally regarded as safe, well established formulation techniques for chemical modification and particulate preparation with polymers, protect the encapsulated antigens from harsh environment, can be pH sensitive and suitable for colon delivery (e.g. Eudragits coating), able to co-deliver immunostimulants and antigens, loading capacity may be limited due to intrinsic chemical properties of polymers and antigens.
Natural polymers	Chitosan, Alginate, Starch, Dextran, Hyaluronic acid, Γ-PGA	Natural resources, generally non-toxic, biocompatible, biodegradable, mucoadhesive and immunostimulating (e.g. chitosan), can work as delivery materials itself or be coated on the surface of other delivery vehicles as mucoadhesive materials.
Lipid based polymers	Liposome (@numerous licensed products), Niosome, Bilosome, Virosome (@Epaxal, Inflexal V), ISCOMs**, Archaeosome	Well established formulation techniques and surface modification, enhance vaccine retention, mucosal sampling, uptake and process by APCs, capable to induce both humoral and cellular immune responses, flexible encapsulation or adsorption of antigens and adjuvants, loading capacity varies as the chemical property like hydrophobicity of antigens and adjuvants changes.
MAP systems	MAP—synthetic peptides	The dendritic scaffold itself (e.g. lysine-based dendrimer) is non-immunogenic and biocompatible, can incorporate multi-epitopes and multifunctional peptides in one system to increase the antigen stability, uptake and immunogenicity.
Hydrogel	Cationic cholesteryl group-bearing pullulan (cCHP)*, GelVac*	Prolong the mucosal clearance, efficiently trap protein antigens in nano-gels; suitable for vaccine lyophilization formulation.
Inorganic particles	Gold	Non-immunogenic, biocompatible and easy fabrication in size and shapes.
Receptor-ligand mediated delivery	UEA-1, RGD peptide, Ganglioside GM1 ligand, Co1, Fc, Mannose, IgG, Transferrin, Claudin-4	Enhance mucosa permeability and increase specific mucosal/immune cell uptake mediated by the receptor-ligand interaction.

The current status in clinical study of the materials are indicated by * (phase 1), ** (phase 2), *** (phase 3), and @ (licensed product with trade names). The table only summarizes the general delivery materials in protein-based vaccines mentioned in this review. More information regarding to the detailed investigation reports can be found in the Table S2

For more details of various delivery systems of vaccines, the reader is referred to other reviews by Rydell et al. on starch (Rydell et al., 2005), by Sahdev et al. on biomaterials (Sahdev et al., 2014), by Tiwari et al. on liposomes (Tiwari et al., 2010), by Hu et al. on ISCOMs (Hu et al., 2001), by Barbara et al. on mucoadhesives (Baudner and O’Hagan, 2010), by Zhao et al. on VLPs (Zhao et al., 2013), by Demento et al. on PAMP-modified biomaterials (Demento et al., 2011).

### Virus-like particles (VLPs)

VLPs with a diameter of 20–100 nm, are pseudo-virons self-assembled from viral envelope or capsid proteins. They display structural characteristics of viruses but is not infectious or replicating due to the lack of virus genes (Scheerlinck and Greenwood, 2008). They are highly immunogenic due to the particular, ordered and repetitive structural characters mimicking the nature of virus. The constitution proteins can be produced with mammalian cells, insect cells, yeast, bacteria and even plants through recombinant DNA techniques (Santi et al., 2008; Zhao et al., 2013).

Bearing these properties, VLPs are exploited as the delivery system for protein/peptide antigens, since they can display multiple epitopes of infectious pathogens on the surface of their constitution proteins via genetic fusion or chemical conjugation. The fused influenza M2 extracellular region and the HBV core protein can be efficiently expressed in *E. coli* and spontaneously form VLPs after purification. These VLPs induce efficient protective immunity against influenza virus in mice after intraperitoneal or intranasal administration (Neirynck et al., 1999; De Filette et al., 2006; Ibanez et al., 2013). The VLP assembled from recombinantly modified capsid proteins of HEV was found to have HIV immunogenicity since it incorporated the P18 peptide sequence from gp120 of HIV, but escaped the pre-existing anti-HEV immunity because the epitopes of the HEV capsid protein was mutated (Jariyapong et al., 2013). The VLP of modular murine polyomavirus (MuPyV) was reported as an efficient antigen delivery system for the peptide antigen of group A streptococcus (GAS) since the fused antigen-VLP successfully induced immune protection in mice upon intranasal vaccination without adjuvants (Rivera-Hernandez et al., 2013). VLPs derived from enteric pathogens like Norwalk virus (NV) was believed to be a promising delivery candidate for oral and intranasal vaccines (Mason et al., 1996; Ball et al., 1998; Guerrero et al., 2001). Although most VLPs do not necessarily need extra adjuvants in the vaccination, researchers have shown that VLPs with immunostimulants such as detoxified CT, VLP-trapped nucleic acids, GM-CSF or CD40 ligand induced better immune protection (De Filette et al., 2006; Skountzou et al., 2007; Ibanez et al., 2013).

VLPs have been licensed and commercialized in diseases caused by hepatitis B virus (HBV) (Krugman, 1982; Scolnick et al., 1984), human papillomavirus (HPV) (Shank-Retzlaff et al., 2005; Deschuyteneer et al., 2010) and hepatitis E virus (HEV) (Li et al., 2001; Wu et al., 2012). Despite the successful usage and potent immunity, VLPs as a delivery system for protein antigens are still limited by the relatively complicated genetic modification on protein fusion and the subsequently required structural integrity characterization. Meanwhile, lipids and proteins from the expression host may also be assembled into VLPs. Thus, various expression systems could generate differential VLPs despite the fact that they are assembled primarily from the same viral protein (Grgacic and Anderson, 2006).

### Particulate systems based on synthetic polymers

#### Polyesters (PLGA, PLA and PCL) based particular systems

PLGA is one of the most successfully adopted biodegradable polymers for therapeutics and vaccine delivery. It embraces several attractive properties: 1) biodegradability and biocompatibility, 2) generally regarded as safe when used in drug delivery system for parenteral administration, 3) well-developed techniques and methodologies to encapsulate drugs with various physiochemical properties, 4) protection of drugs from degradation, 5) controllable surface modification and 6) controllable sustained release.

PLGA microspheres and nanoparticles have been explored as delivery systems for mucosal vaccine development of protein antigens. PLGA microparticles containing OVA elicit sustained OVA-specific humoral and mucosal immunity in cattle after intranasal administration (Kavanagh et al., 2003), and in mice after oral delivery especially when immunostimulant MPLA is co-encapsulated (Sarti et al., 2011). Multiple oral vaccinations of OVA encapsulated in PLGA particles can stimulate CTL immune response in mice, although not as efficient as the formulation of OVA with ISCOMs (Maloy et al., 1994). Mansoor et al. reported that PLGA particles encapsulating bovine parainfluenza virus type-3 (BPI3V) peptides or proteins induced an early, gradually increasing humoral immune responses via intranasal delivery, suggesting the advantage of slow and prolonged release of antigens in particulate systems compared to those in the soluble form (Mansoor et al., 2014). Two peptide antigens of bovine respiratory syncytial virus (BRSV) co-encapsulated in PLGA microparticles induced both mucosal immune response in upper and lower respiratory tract and T-cell mediated immune response in mice after a single-dose intranasal administration, which is not observed with soluble antigens (Kavanagh et al., 2013). Peptide antigens of malaria carried with PLGA microparticles induced stronger systemic immune response compared to those absorbed to alum either orally or subcutaneously administered in mice, and meanwhile the Th1-mediated cellular immune response was only observed in the previous formulation (Carcaboso et al., 2003). In another study, recombinant envelope protein E2 of classical swine fever virus (CSFV-E2) as the antigen was encapsulated in PLGA microspheres, with rabbit serum albumin as the protein stabilizer, for the mucosal and systemic vaccine development (Brandhonneur et al., 2009). Immunization tests were realized in rabbits through three routes (intranasal, oral and intramuscular) followed by an intradermal boost. The response after intranasal administration was found to be more stable and intense than that with the oral route.

Protein/Peptide-based vaccines with F1 or/and V antigens of *Y. pestis* induced higher humoral and mucosal immune response in mice upon intranasal administration using PLGA/PLA microspheres as the delivery system compared to the soluble antigens (Eyles et al., 2000; Alpar et al., 2001; Tripathi et al., 2006). Vaccination through the intranasal route in mice induced and maintained long-lived protective immunity against the challenge of *Y. pestis* (Ulery et al., 2011). The *S. equi* M-like protein (SeM) antigen was encapsulated in PCL nanospheres containing mucoadhesive polymers (chitosan or alginate) or absorption enhancers (spermine or oleic acid) to develop the vaccine against equine infections (Florindo et al., 2009). This vaccine formulation induced higher immune response than the free antigens in mice after intranasal administration. PCL nanoparticles encapsulating DT induced higher DT-specific IgG response in mice after intranasal immunization than PLGA/PLGA-PCL blend/co-polymer nanoparticles, which was attributed to the increased antigen uptake into cells with the more hydrophobic PCL nanoparticles (Singh et al., 2006).

The surface properties of PLGA, PLA and PCL particles can be modified with hydrophilic PEG or positively charged chitosan/PEI to enhance the protein antigen delivery. For example, OVA encapsulated PEGylated PLGA-based nanoparticles prepared from mixed polymers of PLGA, PLGA-PEG and PCL-PEG, was reported to induce OVA-specific IgG response in mice after oral administration (Garinot et al., 2007). Nanoparticles of PEG-PLA-PEG copolymers were reported to be more efficient in the oral delivery of HBsAg and induced higher systemic and mucosal immunity in mice than PLA nanoparticles (Jain et al., 2010). Radio-labeled TT antigens were loaded in PLA or PLA-PEG nanoparticles, for intranasal administration in rats. It was found that the hydrophilic PLA-PEG nanoparticles showed significantly increased stability in mucosal fluids and enhanced mucosa permeability of antigens compared to the hydrophobic PLA nanoparticles (Tobio et al., 1998; Vila et al., 2004). Meanwhile, PLA-PEG nanoparticles performed better as a protein carrier in the antigen transportation than the microparticles (Vila et al., 2005). Chitosan modified PLGA microparticles showed prolonged residence time of particles on the intranasal mucosa compared to the non-modified ones in rabbits. This HBsAg encapsulated system induced humoral, cellular and mucosal immunity in mice after intranasal administration (Jaganathan and Vyas, 2006). Similarly, HBsAg encapsulated and PEI modified PLGA microspheres induced enhanced immune response in mice after pulmonary administration compared to the non-modified ones (Thomas et al., 2009).

#### Polyethyleneimine based systems

Wegmann et al. suggested that PEI microparticles could serve as a potent mucosal and systemic delivery system and an intrinsic adjuvant for viral glycoprotein antigens (Wegmann et al., 2012; Sheppard et al., 2014). PEI was investigated as a protein antigen delivery system to promote cross-presentation through MHC I pathway using the antigen model OVA (Chen et al., 2011). In their studies, robust antibody-mediated protection was induced in mice and rabbits with a single intranasal administration of influenza HA or herpes simplex virus type -2 glycoprotein (HSV-2 gp) using PEI as the nano-carrier and adjuvant (Wegmann et al., 2012).

#### Eudragits based systems

Oral delivery of protein vaccines or drugs targeting gut-associated lymphoid tissues (GALT) needs to surpass the highly acidic and proteolytic environment in the GI tract (Wang et al., 2009). In this case, specific pH-sensitive polymers like Eudragits are often adopted. Eudragits are methacrylate-based polymers designed to dissolve at specific pH ranges depending on the polymer chemistry. Liu et al. reported a Eudragit S100 coated calcium alginate gel beads for sufficient oral protein/peptide drug delivery to target the colon region. Before trapped into the alginate beads, the peptide drug was first loaded into protecting liposomes (Liu et al., 2003). Zhu et al. successfully delivered an HIV Env epitope-based peptide antigen to the large intestine of mice through oral administration (Zhu et al., 2012). This was realized by encapsulating the peptide antigen in micro-sized agglomerates of PLGA nanoparticles followed by granulation with Eudragit FS30D, an enteric polymer that only dissolves at pH above 7.0. Antigens in the Eudragit formulation given orally induced comparable immune protection as the antigens administered colorectally.

### Particulate delivery system based on natural polymers

#### Chitosan

Chitosan and its derivatives have long been evaluated as a suitable mucosal delivery material for protein/peptide drug and gene therapy (Garcia-Fuentes and Alonso, 2012). Recently chitosan based systems were also evaluated as potential adjuvants and vaccine delivery systems for mucosa vaccines (Illum et al., 2001; van der Lubben et al., 2001; Muzzarelli, 2010; Kobayashi et al., 2013). As an immunostimulant, the ability of chitosan in inducing mixed Th1/Th2 immune response is still controversial depending on the administration routes (Shibata et al., 2001; Porporatto et al., 2005; Oliveira et al., 2012). The antigen TT in N-trimethyl chitosan-mono-N-carboxymethyl chitosan (TMC-MCC) complex nanoparticles induced humoral and cellular immune response in mice after intranasal administration (Sayin et al., 2009). Vicente et al. reported a chitosan nanocapsule co-delivery system, with an oily inner core carrying hydrophobic immunostimulant imiqimod and a cationic chitosan corona to absorb anionic HBsAg on the surface. This co-delivery system induced balanced humoral and cellular immune response in mice after intranasal vaccination (Vicente et al., 2013). Several aspects regarding to the properties of chitosan might need to be improved, including the target specificity, the even size distribution and the solubility in physiological environment (Sahdev et al., 2014).

#### Alginate

Alginate is a biocompatible, biodegradable and mucoadhesive polymer, which could also serve as a protein delivery vehicle. Alginate coated chitosan nanoparticles could serve as a potential mucosal vaccine delivery system to prevent loaded protein antigens from enzymatic degradation. Intranasal or oral delivery of recombinant HBsAg using this system with CpG as the adjuvant efficiently elicits humoral mucosal immune responses in mice (Borges et al., 2007; Borges et al., 2008). Alginate microparticles were observed to be effective for protein antigens delivery against respiratory diseases through intranasal administration but not the oral route in cattle (Rebelatto et al., 2001). Chitosan nanoparticles coated with lectinized alginate were found to be an efficient oral delivery system for the antigen BSA in targeting M-cells and successfully induced systemic and mucosal immunity in mice (Malik et al., 2012). Tafaghodi et al. evaluated the dry powder vaccine formulation of TT encapsulated in alginate microspheres for intranasal immunization in rabbits (Tafaghodi and Rastegar, 2010). Their results suggest that alginate microspheres, with QS as the adjuvant, and cross-linked dextran microspheres as an adsorption enhancer, coordinately increase the titers of systemic IgG and mucosal sIgA.

#### Starch

Compared to intranasal immunization, oral vaccination with polyacryl starch microparticles conjugating HSA (Wikingsson and Sjoholm, 2002), DT (Rydell and Sjoholm, 2004) or its non-toxic mutant (Rydell and Sjoholm, 2005) could induce relatively stronger systemic and mucosal antibody response in mice. These results suggested polyacryl starch microparticles as a promising oral adjuvant for protein antigens. Formaldehyde treatment of the DT mutant before conjugation to microparticles induced better immune response than the reversed order (Rydell and Sjoholm, 2005). Unlike other microparticles which only protect protein antigens from degradation, silicone grafted starch was observed to facilitate the release of the encapsulated HSA and to increase the mucosal immunogenicity after oral and intranasal administration in mice (McDermott et al., 1998).

#### Dextran

Dextran, as a complex branched glucan product of microbial fermentation, is mucoadhesive and can act as mucosa permeation enhancer in protein vaccine delivery. For instance, TT encapsulated in cross-linked dextran microspheres (CDM) with the adjuvant CpG induced both potent systemic IgG and mucosal IgA immune response in rabbits after intranasal administration, while the alum absorbed TT failed to induce potent mucosal IgA secretion (Sajadi Tabassi et al., 2008).

#### Hyaluronic acid

Hyaluronic acid (or hyaluronan), a natural component of cartilage, is a linear polysaccharide comprising repeating disaccharide units of D-glucoronic acid and N-acetyl-D-glucosamine. It participates in the immune reaction by modulating the trafficking of leukocytes, the maturation of epidermal DCs and the activation of T-cell in the antigen presentation (Mummert, 2005). It has been explored for protein vaccine delivery due to its excellent safety, biocompatibility, biodegradability, hydrophilicity and muco-adhesiveness (Sahdev et al., 2014). Intranasal administration of influenza HA and the adjuvant of detoxified LT, by using an esterified hyaluronic acid (HYAFF) microsphere system, induced more potent immune response in mice, rabbits and small-pigs than the conventional intramuscular immunization. Meanwhile, the HYAFF microsphere formulation induced higher antibody titers *in vivo* than the soluble mixed antigen and adjuvant (Singh et al., 2001).

#### Gamma-polyglutamic acid

Gamma-polyglutamic acid (γ-PGA) is a high molecular weight polymer of glutamic acid, where the linkage is between the amino group and the carboxylate side chains. It is a natural product of bacterial fermentation and is the major constituent of a traditional Japanese food ‘natto’. It was applied for medicine and vaccine applications due to its good biocompatibility and low cytotoxicity. The hydrophilic γ-PGA can be hydrophobically modified with L-phenylalanine ethyl ester (L-PAE) to produce the amphipathic γ-PGA-L-PAE. OVA-encapsulated nanoparticles of γ-PGA-L-PAE induced OVA-specific antitumor immunity in mice after intranasal vaccination (Matsuo et al., 2011). Although the results showed similar total IgG titers between the immunization with nanoparticle-encapsulated and free vaccines, the antigen delivered with the γ-PGA-L-PAE nanoparticle systems efficiently induced antigen-specific Th1-dominant cellular immune responses in spleens and lymph nodes. Recently, a nanomicelle system self-assembled from cholesterol conjugated γ-PGA derivatives was observed to be able to serve as both mucus delivery and cellular-immunity-inducing adjuvant for the protein antigen OVA (Noh et al., 2013).

### Lipid-based vehicles (liposomes, niosomes, bilosomes, virosomes, archaeosomes and ISCOMs)

Besides the polymeric particulate delivery systems mentioned above, there is another large group of delivery vehicles based on lipids. Lipid microparticles have been reported as a successful intranasal delivery system for introducing mucosal immune response with HBsAg in rats (Saraf et al., 2006). Similar to the above-mentioned polymers, the lipid microparticles could be prepared with soya lecithin with/without stearylamine using double emulsion method. Yet, most lipid-based vehicles were prepared in the form of liposomes for drug and vaccine delivery. According to the source and properties of constituent materials, liposomes could be further named as normal liposomes, niosomes, bilosomes, virosomes, archaeosomes and ISCOMs.

#### Liposomes

Liposomes are artificial vesicles of one or more layers of phospholipids and an internal aqueous core. They have been widely used for the delivery of therapeutics and vaccines due to their proven safety, biocompatibility and ease of manufacturing (Torchilin, 2005). Depending on the hydrophilicity, hydrophilic antigens (proteins, peptides and carbohydrates) can be entrapped within inner aqueous cavity of the liposome, and hydrophobic antigens (lipoproteins and lipophilic adjuvants) can be inserted in the lipid membrane. Besides, antigens and adjuvants can also be conjugated or adsorbed to the liposome surface. Large unilamellar liposomes (100 nm to 1 μm in diameter) are generally more stable than their smaller counterparts (30–100 nm) due to low curvature and less surface tension (Dawidczyk et al., 2014).

DT and TT loaded liposomes induced neutralizing antibody response in rabbits and monkeys after oral administration with the adjuvant alum (Mirchamsy et al., 1996). HA loaded liposome system was used to deliver HBsAg intranasally in mice, which showed higher mucosal uptake, and stronger mucosal and cellular immune responses compared to intranasally administered placebo or intramuscularly administered with the mixed HA and alum system (Tiwari et al., 2011b). Lectin-UEA1 modified liposomes were found to be able to mediate antigen targeting to M cells and lead enhanced systemic and mucosal immune responses in mice after oral or intranasal administration, compared to non-modified ones encapsulating the fluorescent label or the model antigen BSA (Clark et al., 2001; Li et al., 2011a; Li et al., 2011b). IgG-coupled liposomes encapsulating HBsAg were reported to induce both systemic and mucosal immunity through escalating the antigen uptake by M cells upon intranasal vaccination in mice, while the alum-adsorbed HBsAg was unable to induce immune response when administered intramuscularly (Tiwari et al., 2011a). Minato et al. found that the dose of PEG-modified liposomes encapsulating OVA significantly affected the balance between systemic and mucosal immune responses in mice after oral administrations (Minato et al., 2003). This was attributed to different release rates on the intestinal mucosa surface.

Oligomannose-coated liposomes (OMLs) induced significant systemic and mucosal immune responses in mice after intranasal vaccination with encapsulated antigen OVA, and such effect was absent in non-coated liposomes entrapping OVA or OVA alone (Ishii and Kojima, 2010). OMLs encapsulating full-length HA-NA protein (OML-HN) induced viral-specific systemic and mucosal immunity against human parainfluenza viruses (HPIVs) in mice after intranasal vaccination with the presence of poly I:C (Senchi et al., 2013). The synthetic oligomannose was suggested to be a useful mucosal adjuvant since it embraced comparable efficiency as CTB and acted as a possible M cell-targeting mucosal adhesive material (Ishii and Kojima, 2010).

#### Niosomes and bilosomes

The non-ionic surfactant vehicle (NISVs or niosomes), of non-biological origin, is a chemical/biological stable alternative to liposomes. They share similar hydrophathicity properties as liposomes and were explored for protein vaccine delivery. Niosomes were used to deliver glycoprotein B (gBs) or polylysine rich peptide DTK of herpes simplex virus (HSV) intranasally in mice, which successfully induced humoral and cellular immune protection against genital herpes (Cortesi et al., 2013). Niosomes incorporating bile salts are termed bilosomes. Bile salts in the lipid bilayers could stabilize bilosomes against the bile acid in the gastrointestinal tract. The inner aqueous space of bilosomes could entrap vaccine antigens like proteins/peptides for efficient oral delivery, which have been tested on HA (Mann et al., 2009), HBsAg (Shukla et al., 2008; Shukla et al., 2010), DT and TT (Mann et al., 2006; Shukla et al., 2011; Jain et al., 2014). The results indicated that bilosomes could be a potential oral delivery system for these protein antigens, since significant systemic and mucosal immunity were observed in all tested bilosomes. The induced balance of Th1/Th2 response after oral administration could be modulated through physically modifying the size of bilosomes in the delivery vehicle, as evidenced in the study with influenza antigen HA tested in mice and ferrets (Mann et al., 2009).

#### Archaeosomes

Archaeosomes prepared from archaeal lipids is also believed to be a promising mucosal vaccine adjuvant and delivery system to encapsulate protein antigens. Protein antigen OVA encapsulated in this system induced humoral immune responses but no mucosal immunity after intranasal administrations in mice (Patel et al., 2007). The addition of multivalent cations like CaCl_2_ was able to convert the protein antigen loaded archaeosomes (100–200 nm in diameter) into an archaeal lipid mucosal vaccine adjuvant and delivery (AMVAD) system (Patel and Chen, 2010). The AMVAD is a larger, aggregated structure (most with a diameter of less than 5 μm) like a bunch of grapes. OVA encapsulated in the AMVAD system could induce potent, long lasting and antigen-specific humoral, mucosal (local and remote sites) and CTL response in mice after intranasal immunization (Patel et al., 2007; Patel et al., 2008). The AMVAD system was believed to be a relatively safe vaccine delivery system for intranasal administration as tested with 10-fold excess of dose required for vaccine efficacy (Patel et al., 2008). The AMVAD system could be advantageous to other liposomes due to: 1) high loading efficiency for hydrophilic protein antigens both inside the archaeosomes and in-between; 2) increased stability against autoxidation since archaeal lipid consists of saturated side chains instead of the unsaturated forms; 3) no additional immunostimulating adjuvant is required.

#### Virosomes

Virosomes consist of viral surface proteins (e.g. HA and NA from influenza virus) embedded in a lipid membrane with no internal gene materials. Thus they have the cell fusion activity but no replication ability, and can be explored as drug and vaccine delivery vectors. Pederson et al. evaluated the vaccination effect with H5N1 influenza virosomes through sublingual, intranasal and intramuscular administration in mice with the adjuvant c-di-GMP. Both sublingual and intranasal vaccination induced better local mucosal and systemic cellular immune responses than the intramuscular administration, where the response with intranasal route was stronger than that with the sublingual one (Pedersen et al., 2011).

#### ISCOMs

Immune-stimulating complexes (ISCOMs) were documented as both a delivery system and an immunostimulant for the vaccine development. ISCOMs are 40 nm nano-vectors of open cage structure and comprise cholesterol, phospholipids and mixture of saponins extracted from *Quillaja saponaria* Molina (Sanders et al., 2005; Skene and Sutton, 2006; Sun et al., 2009b). Vaccines of ISCOMs induced systemic and mucosal immune responses upon intranasal administration, incorporating protein antigens such as respiratory syncytial virus (RSV) envelope proteins (Hu et al., 1998), *Mycoplasma mycoides* subsp. *mycoides* (MmmSC) prtoein (Abusugra and Morein, 1999), and recombinant HBsAg (Pandey and Dixit, 2010). Initially only hydrophobic antigens like membrane proteins were incorporated in the ISCOMs (Morein et al., 1984), but later ISCOMATRIX was developed to mix with antigens to circumvent the incorporation dependence on antigen properties. ISCOM and ISCOMATRIX can induce humoral and CTL responses (controversial for ISCOMATRIX) (Scheerlinck and Greenwood, 2008). Although the immunity potentiating mechanism is presently unclear, ISCOMs have been suggested as a potential intranasal and oral vaccine delivery system for protein antigens (Mowat et al., 1999; Hu et al., 2001).

### Other miscellaneous vaccine delivery systems

#### Multiple antigen-presenting vaccine systems

Tam developed the multiple antigenic peptide (MAP) system using a non-immunogenic lysine-based dendritic scaffold to improve the immunogenicity of subunit peptide vaccines (Tam, 1988). The MAP system was found to be able to increase the stability, uptake and immunogenicity of those conjugated peptide antigens. To further improve the immunogenicity, components of various functions are incorporated into the MAP, such as helper T-cell epitopes, immunostimulant lipid moieties and cell-penetrating peptides (Fujita and Taguchi, 2011). Ali et al. reported F1-MAP (the B and T epitopes of F1antigen carried by MAP) encapsulated in PLGA microspheres induced strong humoral and mucosal immune responses in mice after the intranasal vaccination, which was significant higher than that with mixed short epitope peptides without conjugation to the MAP. They also found that the co-administration of the encapsulated adjuvant CpG significantly increased the immunity of F1-MAP against plague infections (Ali et al., 2013).

#### Hydrogels

Nano-sized hydrogel of cholesteryl-group-bearing pullulan (CHP) can trap protein drugs or antigens through non-aggregating hydrophobic interaction and gradually release the native drugs or antigens from its polymer network. Nochi et al. and Kong et al. reported a cationic CHP (cCHP) nano-gel as a successful adjuvant-free intranasal vaccine carrier for protein antigens (BoHc/A, TT and PspA), which was proved to induce antigen-specific immune protection against infectious diseases in mice. Both groups found no vaccine invasion into olfactory bulbs or the central nervous system after intranasal vaccination, suggesting cCHP nanogels as an effective and safe intranasal vaccine delivery system for protein antigens (Nochi et al., 2010; Kong et al., 2013). An inert *in situ* gelling polysaccharide (GelSite) extracted from *Aloe vera* was used in a dry-powder vaccine formulation (GelVac) for intranasal delivery of Norwalk VLPs (Velasquez et al., 2011). In this study, GelVac formulation delayed the mucociliary clearance and prolonged antigen exposure to the immune effector sites due to the *in-situ* gelation at the nasal mucosa.

#### Gold nanoparticles

Gold nanoparticles are used in delivering subunit vaccines without inducing anti-gold antibodies (Chen et al., 2010). They could promote immune response via different cytokine pathways depending on the size and shape (Niikura et al., 2013). The vaccine of TT loaded in chitosan-functionalized-gold nanoparticles, with *Quillaja saponaria* extract as the adjuvant, induced 28-fold immune response in mice after oral administration compared to the one without using nanoparticles as the delivery system (Barhate et al., 2013). The vaccine of extracellular domain of M2 (M2e) conjugated to gold nanoparticles with the adjuvant CpG induced protective immune response against influenza A virus after intranasal administration in mice (Tao et al., 2014).

### Receptor-ligand mediated vaccine delivery systems

Many receptors on the mucosal epithelial cells, M cells and APCs have been explored for vaccine target-delivery with antigens co-delivered with specific receptor-binding ligands, such as lectins, bacterial adhesins, bacterial toxins, PAMPs, other M-cell targeting ligands, antibodies and Fcs (Takahashi, 2003; Sneh-Edri et al., 2011; Cruz et al., 2012; Devriendt et al., 2012). Similarly, receptors on DCs have been targeted with DC-targeting proteins fused with cancer antigen protein in cancer immunotherapy (Ma et al., 2014b). These ligands can be fused to antigens directly or conjugated/absorbed to the surface of the delivery system (Ma et al., 2014b).

For example, CD71 is a highly efficient transcytotic and recycling transferrin receptor located on the nasal and vaginal mucosal epithelium. Thus, transferrin was utilized as a drug/vaccine conjugate to specifically target CD71 (Qian et al., 2002; Mann et al., 2012). Transferrin conjugation to a model HIV-1 trimeric gp140 antigen endowed the vaccine with efficient mucosal targeting, especially through the intranasal administration (Mann et al., 2012). Claudins are considerably expressed on tumor cells and mucosal epithelium cells, thus they have been explored for targeting delivery in tumor therapy and mucosal vaccination. OVA fused to claudin-4 binding ligands induced both Th1- and Th2-mediated immune response in mice, suggesting clauding-4 targeting as an effective way for intranasal vaccination (Nagase et al., 2013). Ad2F, an epithelial cell binding domain, was fused to a botulinum neurotoxin A (BoNT/A) immunogen-Hcbtre (Staats et al., 2011). The fused Ad2F-Hcbtre induced higher antibody response in rabbits after intranasal immunization compared to the non-fused Hcbtre antigen alone.

Lectins such as UEA-1 can mediate mucosal targeting delivery through binding to their receptors on the apical membranes of M cells. Lectinized PLGA particles encapsulating HBsAg enhanced the immune response after oral administration compared to non-lectinized ones (Gupta et al., 2007). CKS9 is an M-cell homing peptide identified with phage display technique, which significantly increased the M-cell mediated uptake of CKS9-surface modified chitosan nanoparticles (CKS9-CS) (Yoo et al., 2010). PLGA nanoparticles coated CKS9-CS significantly enhanced the systemic and mucosal immune response of BmpB (protein antigen of swine dysentery) vaccine after oral administration (Jiang et al., 2014). RGD peptides were displayed on vaccine delivery system like PEGylated PLGA nanoparticles to target apical side β-integrin of M cells using oral administration in mice (Garinot et al., 2007). Other M-cell targeting ligands, such as GM1 ganglioside and Co1, have also been reported to induce increased systemic and mucosal immune response for fused protein antigens after oral administration in mice compared to the non-fused antigens (Kim et al., 2006; Kim et al., 2010).

Protein Ag fused to IgG or Fc can specifically target IgG/Fc-receptors constitutively expressed on APCs to enhance humoral and cellular immunity (Nimmerjahn and Ravetch, 2008; Gosselin et al., 2009). PLGA nano-/micro-particles were surface-modified with DC-targeting antibodies and peptides to improve antigen presentation (Lewis et al., 2012). Du et al. and Ma et al. successfully vaccinated mice by inducing strong systemic antibody response through intramuscular/subcutaneous administration of a recombinant protein containing receptor-binding domain (RBD) of SARS-/MERS-CoV spike protein fused with Fc of IgG (RBD-Fc) (Du et al., 2007; Du et al., 2013b). Later, they reported that much stronger local mucosal immune response was induced with intranasal vaccination with the same RBD-Fc antigen than subcutaneous immunization (Ma et al., 2014a). The RBD fusion with Fc also conferred the RBD-Fc with dimeric conformation (Du et al., 2013a; Ma et al., 2014a), which may improve the immunogenicity.

## CONCLUSIONS AND PERSPECTIVES

Mucosal vaccine with protein antigens is a very promising product format for future vaccine development, especially considering the desired immunity protection at the mucosal surface and the safety of protein antigens. However, the low immunogenicity and weak stability of free protein antigens in the relatively harsh mucosal fluid (such as nasal mucus and gastric conditions), require an optimized vaccine formulation to enhance immune protection. A wide range of particulate delivery systems have been proven to be efficient in vaccine delivery in animals and human. Co-administration of suitable immunostimulants with protein antigens could facilitate and enhance the generation of potent antigen-specific immune protection. Therefore, a successfully designed vaccine therapeutics is always an optimal combination of antigens, immuno-potentiators, vaccine carriers, and an effective formulation of the above, which is delivered through an appropriate administration route.

Current research suggested that mucosal delivery of properly designed formulation of protein-based antigens could provide efficient humoral protection in animal models. Nontheless, the detailed mechanism through which different vaccine therapeutics actually generate and enhance the vaccination protection is still awaiting to be investigated, which could be realized by systemic comparation of the effect of various formulations and administation routes on the same antigen. More specificly, how different delivery carriers and immunostimulants, and their properties such as size, geometry, *in vivo* kinetics and molecular patterns contribute to the mucosal sampling, the vaccine uptake, process, presentation and finally lead to the desired immunity? Meanwhile, more research efforts are required to design mucosal vaccines to induce strong cellular immunity, including the Th1 and CTL responses, especially for diseases like tuberculosis, HIV and malaria where cellular immunity is crucial in mediating protection.

## Electronic supplementary material

Supplementary material 1 (PDF 339 kb)

## ABBREVIATIONS

APC: antigen presenting cell
BSA: bovine serum albumin
β-gal: β-galactase
CoV: coronal virus
CpG: unmethylated deoxycytidyl-deoxyguanosine oligonucleotide
CT: cholera toxin
DC: dendritic cell
DT: diphtheria toxoid
EGFP: enhanced green fluorescent protein
gp: glycoprotein
γ-PGA: γ-polyglutamic acid
HA: hemaggluttinin
HBsAg: hepatitis B surface antigen
HBV: hepatitis B virus
HEV: hepatitis E virus
HIV: human immunodeficiency virus
HPV: human papillomavirus
IFN-γ: interferon-γ
IL-12: interleukin-12
ISCOM: immune-stimulating complexes
LPS: lipopolysaccharide
LT: heat-labile enterotoxin
MALT: mucosa-associated lymphoid tissue
MAP: multiple antigenic peptide
M cell: microfold/membraneous cell
M protein: matrix protein
MCC: mono-N-carboxymethyl chitosan
MERS: middle east respiratory syndrome
MPLA: monophosphoryl lipid A
NALT: nasal-associated lymphoid tissue
NA: neuraminidase
OVA: ovalbumin
PAMP: pathogen-associated molecular pattern
PCL: poly ε-caprolactone
PEG: polyethyleneglycol
PEI: polyethyleneimine
PLA: poly lactic acid
PLGA: poly lactic-co-glycolic acid
poly I:C: polyriboinosinic-polyribocytidylic acid
PP: Payer’s Patch
PSA: pig serum albumin
QS: Quilaja saponin
RSV: respiratory syncytial virus
rUre: recombinant *H. pylori* urease
SARS: severe acute respiratory syndrome
STING: stimulator of interferon genes
Th1/2/17 cell: T helper 1/2/17 cell
TLR: Toll-like receptor
TMC: N-trimethyl chitosan
TT: tetanus toxoid
VLP: virus-like particle
