# Supplementary material for: Intranasal and oral vaccination with protein-based antigens: advantages, challenges and formulation strategies
Source: Protein Cell. 2015 May 6;6(7):480–503. doi: 10.1007/s13238-015-0164-2 (PMC4491048; doi:10.1007/s13238-015-0164-2)
Supplement: Supplementary file 1 — Supplementary material 1 (PDF 339 kb) [file 13238_2015_164_MOESM1_ESM.pdf]

Supplementary table 1. List of promising adjuvants tested for intranasal and oral vaccination with protein antigens

Adjuvants	Administration /Animals	Protein antigens	Reported responses	References
**Alum**				
Alum	Oral/rabbits, monkeys	DT, TT	IgG1,Th2	Mirchamsy et al. 1996
**Bacterial toxins and their derivatives**				
Cholera toxin	Nasal/mice	SeM	IgG, IgA, Th1, Th2	Florindo et al. 2009
		M2e-HBc(VLP)	IgG, Th1, protection	De Filette et al. 2006
		rUre	IgG, IgA, Th1, Th2	Moschos et al. 2004
		HA	N.A	Ichinoe et al. 2005
Heat-labile enterotoxin	Nasal/mice, rabbits, pigs	HA	IgG, IgA	Singh et al. 2001
	Nasal/rabbits	HagB	IgG, IgA,	Zhang et al. 2003
**Bacterial glycolipids**				
MPL/LPS	Oral, nasal/mice	OVA	IgG, IgA, Th1, Th2	Sarti et al. 2001, Bal et al. 2012
**Bacterial peptidoglycan**				
MDP	Nasal/mice	OVA	IgG1, IgA	Bal et al. 2012
**Bacterial second messengers**				
c-di-AMP	Nasal/mice	β-Gal, OVA	IgG, IgA, Th1/2/17, CTL	Ebensen et al. 2011
		rNP	IgG, IgA, Th1	Sanchez et al. 2014
c-di-IMP	Nasal/mice	β-Gal, OVA	IgG, IgA, Th1/2/17, CTL	Libanova et al. 2010
c-di-GMP	Nasal/mice	β-Gal, OVA	IgG, IgA, Th1, CTL	Ebensen et al. 2007
		HA	IgG, IgA, Th1, Th2	Svindland et al. 2013
	Nasal, sublingual/mice	H5N1 virosomes	IgG, IgA, Th1, Th2, Th17	Pedersen et al. 2011
**Synthetic bacterial DNA and viral dsRNA**				
CpG	Nasal/mice	HBsAg	IgG, IgA, Th1, Th2, CTLs	McCluskie et al. 1998,
	Nasal/mice	HIV peptides	IgG, IgA	Pun et al. 2009, Buffa et al. 2012
	Oral/mice, rats	HBsAg, TT	IgG, IgA, Th1, Th2, CTLs	McCluskie et al. 2000, Eastcott et al. 2001, Buffa et al. 2012
	Nasal/mice;	SHIV-VLP	IgG, IgA, CTL	Kang et al. 2004
Poly I:C	Nasal/mice	HA	IgG, IgA, protection	Ichinoe et al. 2005
		HPIV-HN	IgG, IgA	Senchi et al. 2013
**Virosomes**				
Virosomes	Nasal/mice	SHIV-VLP	IgG, IgA, CTL	Kang et al. 2004
**Synthetic small organic molecules**				
Imidazoquinoline	Nasal/mice	HBsAg	IgG, Th1, Th2	Vicente et al. 2013
C48/80	Nasal/rabbits	BoNT/A-Hcbtre	IgG	Staats et al. 2011

	Nasal/mice	HA	IgG, IgA, Th1, Th2, protection	Meng et al. 2011
Vitamin E TPGS,	Nasal/mice	DT	IgG	Somavarapu et al. 2005
α-GalCer	Nasal, oral /mice;	HIV-gp120 epitopes	Cellular	Courtney et al. 2009
**Plant derived molecules**				
Saponin	Nasal/mice	HagB	IgG, IgA, Th1, CTLs	Zhang et al. 2003
	Nasal/rabbits	TT	IgA, IgG	Moghadam et al. 2012
Eurocine	Nasal/mice	HA	IgG, IgA, protection	Petersson et al. 2010
**Polymers from crustaceans' shell or particles from surf clam**				
Chitosan	Nasal/mice	M1	IgG, IgA, protection	Sui et al. 2010
		rUre	IgG, IgA, Th1, Th2, cellular	Moschos et al. 2004
		HA	IgG, IgA, Th1, Th2	Svindland et al. 2013
Surf clam microparticles	Nasal/mice	HA	IgG, IgA	Ichinohe et al. 2006
**Cytokines**				
IL-1	Nasal/mice	HA	IgG, IgA, protection	Kayamuro et al. 2010
IL-12	Nasal/mice	TT	IgG, IgA, Th1, Th2	Boyaka et al. 1999
TNF and mutants	Nasal/mice	OVA	IgG, IgA, Th2	Kayamuro et al. 2009a, 2009b

Supplementary table 2. List of materials and delivery vehicles tested for intranasal and oral vaccination with protein antigens

Materials	Administration/Animals	Protein antigens	Reported responses	Delivery vehicles* and adjuvant if applicable	References
**VLP**					
VLP-HBV	Nasal/mice	M2e	IgG, Th1, protection	NPs, CT	De Filette et al. 2006
VLP-HEV	Oral/mice	HEV	IgG, IgA	NPs	Li et al. 2001, Wu et al. 2012
VLP-MuPyV	Nasal/mice	GAS-J8i peptide	IgG, IgA	NPs	Rivera-Hernandez et al. 2013
VLP-NV	Oral, nasal/mice	rNV-VLP	IgG, IgA	NPs, LT/CT	Mason et al. 1996, Ball et al. 1998, Guerrero et al. 2001
**Synthetic polymers**					
PLGA	Nasal, oral/mice, rabbits, cattle	F1/V	IgG, IgA	MPs/MSs	Jaganathan et al. 2006
	Oral/mice	OVA	IgG, IgA	NPs, MPLA	Sarti et al. 2011
		OVA	IgA, CTL	MPs	Maloy et al. 1994
		Malaria-SPf66	IgG, Th1	MPs	Carcaboso et al. 2003
	Nasal, oral/rabbits	SFV-Env E2	Ig, protection	MSs	Brandhonneur et al. 2009
	Nasal/mice	HBsAg	IgA, cellular	CS-PLGA MSs, CTB	Jagananthan et al. 2006
		BRSV-peptides	IgA, cellular	MPs	Kavanagh et al. 2013
		BP13V-peptides	IgG	NPs	Mansoor et al. 2014
	Nasal/calves	OVA	IgG, IgA	MSs/MPs	Kavanaagh et al. 2003
PLA	Nasal/mice	F1/V/(F1+V)	IgG, IgA	MSs	Eyles et al. 2000, Alpar et al. 2001, Tripathi et al. 2006
	Nasal/rats	TT	IgG, IgA	PEG-PLA NPs/MPs	Tobio et al. 1998, Vila et al. 2004
	Oral/mice	HBsAg	IgG, IgA, Th1	PEG-PLA-PEG NPs	Jain et al. 2010
PCL	Oral/mice	OVA	IgG	RGD peptide, NPs of PLGA/PLGA-PEG/PCL-PEG	Garinot et al. 2007
	Nasal/mice	DT	IgG	NPs, VE-TPGS	Somavarapu et al. 2005, Singh et al. 2006
		SeM	IgG, IgA, Th1, Th2	NSs, CTB	Florindo et al. 2009
PEI	Nasal/mice, rabbits	HA, HSV-gpD,	IgG, IgA, cellular, protection	Nanoscale complexes	Wegmann et al. 2012

		HIV-gp140			
Eudragits	Oral/mice	HIV Env epitopes	Colon-targeted, IgG, IgA, cellular, protection	NPs/MPs, TLR ligands	Zhu et al. 2012
	Oral/human	Bee venom peptide	Colon-targeted	Alginate-liposome	Liu et al. 2003
**Natural polymers**					
Chitosan	Nasal/mice	TT	IgG, Th1, Th2	NPs	Sayin et al. 2009
		HBsAg	IgG, Th1, Th2	Nanocapsules, Imiquimod	Vicente et al. 2013
Alginate	Nasal, oral/mice	HBsAg	IgG, IgA, cellular	Alginate-chitosan NPs, CpG	Borges et al. 2007,2008
	Nasal/calves	PSA	IgG	MPs	Rebelatto et al. 2001
	Oral/mice	BSA	IgG, Th1, Th2	Lectin-alginate NPs	Malik et al. 2012
Starch	Oral/mice	DT and its mutant	IgG, IgA, Th1, Th2	Polyacryl starch MPs	Rydell et al. 2004<br>Rydell et al. 2005
		HSA	IgG, IgA, Th1, Th2	Polyacryl starch MPs	Wikingsson et al. 2002
	Nasal, oral/mice	HSA	IgG, IgA, Th2	Silicone-starch MPs	McDermott et al. 1998
Dextran	Nasal/rabbit	TT	IgG, IgA	MSs	Sajadi et al. 2008
Hyaluronic acid	Nasal/mice, rabbits, pigs	HA	IgG, IgA	HYAFF MSs, LT	Singh et al. 2001
Γ-PGA	Nasal/mice	OVA	IgG, IgA, Th1, CTL	NPs	Matsuo et al. 2011<br>Noh et al. 2013
**Lipid based polymers**					
Liposome	Oral/mice	BSA	IgG, IgA	UEA1-liposome	Li et al. 2011a, 2011b
		OVA	IgG, IgA	PEG-liposome	Minato et al. 2003
	Nasal/mice	HBsAg	IgG, IgA, Th1	IgG-liposome	Tiwari et al. 2011a
			IgG, IgA, Th1	HA-liposome	Tiwari et al. 2011b
		OVA	IgG, IgA	Oligomannose-liposome	Ishii et al. 2010
		HPIV-HN	IgG, IgA	Oligomannose-liposome, Poly I:C	Senchi et al. 2013
	Oral/rabbits, monkeys, human	DT and TT	IgG	LRS	Mirchamsy et al. 1996
Niosome	Nasal/mice	HSV-gBs	IgG, Th1, Th2	N.A.	Cortesi et al. 2013
Bilosome	Oral/mice	HA	IgG, Th1, Th2	N.A.	Mann et al. 2009
		HBsAg	IgG, IgA	CTB-bilosome	Shukla et al. 2008, 2010
		TT	IgG, IgA, Th1	N.A.	Mann et al. 2006, Jain et al. 2014
		DT	IgG, IgA	N.A.	Shukla et al. 2011
Virosome	Nasal/sublingual	HA	IgG, IgA, Th1, Th2, Th17	c-di-GMP	Pedersen et al. 2011
ISCOMs	Nasal/mice	RSV Env	IgG, IgA, Th1, Th2	N.A.	Hu et al. 1998
		MmmSC	IgG, IgA, Th1, Th2, CTL	N.A.	Abusugra et al. 1999
		HBsAg	IgG, IgA, Th1, Th2, CTL	N.A.	Pandey et al. 2010

Archaeosome	Nasal/mice	OVA	IgG, IgA, Th1, Th2, CTL	AMVAD	Patel et al. 2007, 2008
**Multiple antigen-presenting systems-synthetic peptides**					
MAP		MAP-F1	IgG, IgA, Th1, Th2	PLGA MPs, CpG	Ali et al. 2013
**Hydrogel of cationic cholesteryl group-bearing pullulan (cCHP)**					
cCHP	Nasal/mice	BoHc/A, TT	IgG, IgA,	Nanogel	Nochi et al. 2010
		PspA	IgG, IgA, Th2, Th17	Nanogel	Kong et al. 2013
**Inorganic particles**					
Gold	Oral/mice	TT	IgG, IgA	CS-AuNPs, QS	Barhate et al. 2013
	Nasal/mice	M2e	IgG, Th1, Th2	M2e-AuNP, CpG	Tao et al. 2014
**Receptor-ligand mediated target delivery**					
UEA-1	Oral/mice	HBsAg	IgG, IgA, Th1, Th2	UEA1-PLGA NPs	Gupta et al. 2007
		BSA	IgG, IgA	UEA-liposome	Li et al. 2011a, 2011b
RGD peptide	Oral/mice	OVA	IgG	PEG-PLGA NPs	Garinot et al. 2007
Ganglioside GM1 ligand	Oral/mice	EGFP	IgG, IgA, Th2	N.A.	Kim et al. 2006
Co1	Oral/mice	EGFP	IgG, IgA, Th2	N.A.	Kim et al. 2010
Fc	Nasal/mice	MERS-RBD	IgG, IgA, Th1, Th2	Fc-RBD, poly I:C	Ma et al. 2014a
Mannose	Nasal/mice	OVA	IgG, IgA	Liposome	Ishii et al. 2010
		HPIV-HN	IgG, IgA	Liposome, poly I:C	Senchi et al. 2013
IgG	Nasal/mice	HBsAg	IgG, IgA, Th1	IgG-liposome	Tiwari et al. 2011a
Transferrin	Nasal/mice	HIV-1 gp140	IgG, IgA	Tf-gp140	Mann et al. 2012
Claudin-4	Nasal/mice	OVA	IgG, IgA, Th1, Th2	OVA-ligands	Nagase et al. 2013

*: MPs: microparticles; NPs: nanoparticles; MSs: Microspheres; NSs: Nanospheres.
